# Immune Responses in Lung Granulomas during Mtb/HIV Co-Infection: Implications for Pathogenesis and Therapy

**DOI:** 10.3390/pathogens12091120

**Published:** 2023-09-01

**Authors:** Deepak Kaushal, Dhiraj K. Singh, Smriti Mehra

**Affiliations:** Southwest National Primate Research Center, Texas Biomedical Research Institute, San Antonio, TX 78227, USA

**Keywords:** TB/SIV co-infection, LTBI, reactivation, ART, chronic immune activation, type I IFN, IDO

## Abstract

HIV and TB are the cause of significant worldwide mortality and pose a grave danger to the global public health. TB is the leading cause of death in HIV-infected persons, with one in four deaths attributable to TB. While the majority of healthy individuals infected with *M. tuberculosis* (Mtb) are able to control the infection, co-infection with HIV increases the risk of TB infection progressing to TB disease by over 20-fold. While antiretroviral therapy (ART), the cornerstone of HIV care, decreases the incidence of TB in HIV-uninfected people, this remains 4- to 7-fold higher after ART in HIV-co-infected individuals in TB-endemic settings, regardless of the duration of therapy. Thus, the immune control of Mtb infection in Mtb/HIV-co-infected individuals is not fully restored by ART. We do not fully understand the reasons why Mtb/HIV-co-infected individuals maintain a high susceptibility to the reactivation of LTBI, despite an effective viral control by ART. A deep understanding of the molecular mechanisms that govern HIV-induced reactivation of TB is essential to develop improved treatments and vaccines for the Mtb/HIV-co-infected population. We discuss potential strategies for the mitigation of the observed chronic immune activation in combination with both anti-TB and anti-retroviral approaches.

## 1. Introduction

Despite the advent of COVID-19, the tuberculosis (TB) and human immunodeficiency virus (HIV) co-pandemic continues to be a major healthcare issue in resource-limited countries [1]. HIV and TB co-infection predisposes the host to the reactivation of latent tuberculosis infection (LTBI), resulting in the worsening of the disease conditions, and in mortality, although a very small number of individuals with LTBI experience reactivation following HIV co-infection [2,3]. The reason why some individuals reactivate while others do not is not well understood and needs to be clarified. Understanding the molecular and immunological mechanisms that mediate reactivation during co-infection will lead to the development of better therapeutic and vaccination approaches for the affected population.

Immune response dysregulation due to HIV and SIV infections. Advances over the past two decades, using both patient-derived samples as well as macaque models of pathogenic infection with SIV, have contributed to our understanding of the immune responses to these lentiviruses. Most HIV infections are mucosal in nature and can be modelled in macaques via the experimental inoculation of SIV intravaginally or intrarectally. It is very likely that mucosal CD4^+^ T cells that also express the coreceptor CCR5 are the first cells infected by the virus [4,5]. From the mucosal surface, the virus is transmitted to the draining lymph nodes and other lymphoid tissues, e.g., local tissue-associated lymphoid tissue, which provide significantly more target cells for the virus to rapidly multiply [4,6]. Dendritic cells (DCs) [7] and B cells [8] may also play a role in the transmission/replication of the virus. Not only a large number of CD4^+^ T cells become infected by the virus, but also even greater proportions of cells undergo rapid cell death, resulting in immune dysregulation [9]. The result of this process is that >90% of all CD4^+^ T cells in tissues are impacted by HIV/SIV infection within the first three weeks, which leads to a profound immunodeficiency. This is true in Mtb/HIV co-infections modeled in NHPs [10,11]. Concomitantly, as the CD4^+^ T cell levels decline, the plasma viral loads increase in the first 3–4 weeks in both NHP models and humans [12]. Eventually, the total levels of CD4^+^ T cell recover, primarily in the peripheral blood, but this recovery is incomplete in both the gut-associated lymphoid tissues of HIV-infected humans and SIV-infected rhesus macaques and the bronchi of Mtb/SIV-co-infected rhesus macaques [5,10,11,13,14,15].

The nonhuman primate (NHP) model of TB and Mtb/HIV co-infection. Nonhuman primates (NHPs) such as rhesus or cynomolgus macaques are excellent models of Mtb infection and TB disease, mimicking important aspects of these conditions, such as a long-term persistent infection with Mtb and a granulomatous pathology. We generated rhesus macaque (RM) models of Mtb infection by exposing the animals to true aerosols of Mtb, thus mimicking the natural route of exposure. A number of research groups, including ours, are currently engaged in investigating the TB granuloma in great detail, using a single-cell, omics, and imaging techniques. NHPs are also excellent models of TB reactivation following HIV co-infection with simian immunodeficiency virus (SIV) as a surrogate for HIV. These are critical models to study the responses to both pathogens in the lung, and even more so, in lung granulomas. This is due to the fact that most data from humans co-infected with Mtb/HIV derive from peripheral blood samples (plasma, sera and PBMCs), as it is difficult to access lung granulomas in these patients over time. We utilized ART [16] to effectively inhibit SIV replication in the periphery as well as in tissues and recapitulate the spectrum of human tissue-specific and clinical outcomes, thus allowing for detailed longitudinal and mechanistic studies that are not possible in humans. Scanga and colleagues also developed co-infection models in NHPs (cynomolgus macaques), which were infected first with SIV and then with Mtb, and successfully introduced ART in these animals [17,18]. Together, however, the data provided by these two groups are limited, and information is still mostly available for the mono-infection.

Studies using the NHP model of *Mtb*/SIV co-infection revealed protective CD4^+^ T cell-independent immune responses that suppress LTBI reactivation. In particular, chronic immune activation rather than mere depletion of CD4^+^ T cells is the key correlate of reactivation due to SIV co-infection. The initiation of combinatorial antiretroviral therapy (cART, ART) was shown to enhance survival and lead to a better control of viral replication, significantly reducing the immune activation in the periphery and lung vasculature in Mtb/SIV co-infected RMs. Robust CD8^+^ T effector memory responses with increased cell proliferation in the lymph nodes and lung tissue were observed in co-infected RMs. However, skewed CD4^+^ T effector memory responses and other signatures of chronic immune activation could persist despite ART.

Chronic immune activation due to HIV/SIV infection and co-infection with Mtb. While the impact of HIV on CD4^+^ T cell depletion in the lymphoid tissues is well characterized [5,13], NHP studies using the Mtb/SIV co-infection model revealed protective CD4^+^ T cell-independent immune responses that suppressed the reactivation of LTBI [10]. These include the promotion of proliferating memory CD8^+^ T cells and the greater induction of bronchus-associated lymphoid tissue [19], which is required for protection from Mtb [20,21] as well as other respiratory diseases. However, the most direct evidence for a CD4^+^ T cell-independent role for the control of Mtb infection in its latent form derived from experiments that demonstrated that macaques with Mtb infection/LTBI also presented chronic immune activation after SIV co-infection, while macaque with Mtb infection/LTBI, which experienced comparable levels of CD4^+^ T cell reduction after administration of a depleting antibody, did not. These results suggest that chronic immune activation resulting from HIV/SIV infection, rather than the mere depletion of CD4^+^ T cell due to HIV/SIV infection, is correlated with reactivation. These results are supported by subsequent findings from other researchers [22]. Chronic immune activation characterized by immune dysfunction is a defining characteristic of chronic HIV and SIV infection. Infection with HIV in humans or with SIV in macaques leads to damage of the mucosal barrier of the gastrointestinal tract, causing an increased translocation of microbial products and a significant decrease in activated memory mucosal CD4^+^ T cells at this anatomical site [6,23]. Immune activation results from the inability of intestinal macrophages to bind to and clear the translocated microbial products [23], which likely leads to an influx of interstitial, immature macrophages to the site of infection and to increased macrophage turnover [24].

Based on the available data, we suggest that type I IFN signaling is an important component of SIV(HIV)-induced chronic immune activation. Indoleamine 2,3, dioxygenase (IDO) inhibits the effective immunity to TB in NHPs [25,26]; these results are supported by observations in patient cohorts across the globe [27,28,29,30]. IDO catabolizes the essential amino acid tryptophan (Trp) to kynurenine (Kyn) [31] and exerts immunosuppression via multiple mechanisms [32,33,34]. IDO expression is induced in the lungs of Mtb-infected RMs [35], on the myeloid-rich inner ring of lung granulomas [25]. The inhibition of IDO activity improved the immune responses and reduced the progression to TB disease in Mtb-only infected RMs [26,36]. Single-cell RNAseq [37,38] and imaging approaches [27] recently revealed the highly immunosuppressed nature of the human TB granuloma microenvironment, which potentially impacts both immune responses and efforts to treat TB. These studies found that IDO was the most abundantly expressed protein in human TB granulomas, thus strongly validating our NHP results. Thus, IDO is a major mediator of the immunosuppression in the TB granulomas and therefore a host-directed therapy (HDT) target. IDO blockade concurrent with TB therapy better cleared Mtb infections in RMs [36]. Since HDT for TB is addressed at individuals with DR-TB or HIV co-infection, IDO is a critical Mtb/HIV-co-infection HDT target. Since IDO can be induced by type I IFN, we propose that the type I IFN–IDO nexus plays an important role in the suppression of productive immune responses during Mtb/HIV co-infection. We will discuss potential strategies for the mitigation of chronic immune activation in combination with to both anti-TB and anti-retroviral approaches.

Granulomas and the immune response to TB. Infection with Mtb leads to the formation of a granuloma—a hallmark of TB that determines the outcome of the infection [39]. Granuloma formation is an iterative process that takes 3–4 weeks [40,41]. In RMs, by week 4, granulomas are complete and comprise granulocytes, primarily neutrophils, which are the first cell type responding to the infection, and interstitial macrophages, which are recruited to the lung parenchyma. Eventually, adaptive immune responses, particularly, associated with antigen-specific T and B cells, are activated in the granuloma. These granulomas may necrose in the core, resulting in a caseated appearance. Granuloma are sites of critical host–Mtb interactions, the result of which leads to either an effective control of the infection, inducing in a quiescent state (latent TB infection or LTBI), or the development of active TB disease. It is believed that granulomas locally help contain the infection, although the specific mechanisms by which they exert an immune control of Mtb, e.g., the spatial understanding of the granuloma function, are not completely understood [42]. This is especially problematic, given that the architecture and composition of granulomas can directly impact both the phenotype of the pathogen and the host immune response, thus affecting the disease outcome in many different ways.

TB granulomas are highly immunosuppressed. Results from the NHP model of TB revealed that Mtb can persist for long periods in granulomas [43]. In the RM model, animals infected with Mtb CDC1551 can be asymptomatic for 7–8 months, when, upon SIV co-infection, they exhibit signatures of LTBI reactivation [43]. While longer experiments with NHPs in an ABSL3 setting are difficult to perform, this result suggests that after a low-dose Mtb infection, the pathogen may indeed persist in a latent form for very long periods in the human lungs. Results from the RM model further provided evidence for this: tubercle bacilli contained within the granulomas of RMs with asymptomatic or LTBI-like infection expressed signatures characterized by dormancy, survival and persistence genes [44]. The recent availability of single-cell approaches has the potential to improve our understanding of granulomas. Single-cell-based approaches are now being used to study gene expression to better understand the TB lung [37,38,45]. Single-cell imaging techniques are being similarly used to study protein expression in these granulomas. These techniques leverage both opportunistically available human granuloma samples as well as samples from experimentally infected animals [27,37] and provide a significantly more detailed picture of granuloma gene expression, cellular composition and function. Single-cell approaches that study gene [37,38,46] and protein expression [27] have revealed the highly immunosuppressed nature of the human TB granuloma [27]. These lesions are depleted of IFN-γ^+^ cells but enriched in TGF-β and IDO^+^PD-L1^+^ myeloid cells. IDO1 (indoleamine 2,3, dioxygenase, IDO), which catabolizes tryptophan (Trp), an essential amino acid, to kynurenine (Kyn) [31], is the most abundant protein in human TB lesions [27]. IDO exerts immunosuppression by multiple mechanisms, e.g., by impacting T cell proliferation in the absence of Trp and, indirectly, via the generation of various products, e.g., Kyn, that are strong inhibitors of both T cell and macrophage function [32,33,34].

TB granulomas express IDO, which induces immunosuppression. New TB granulomas in RMs express a robust pro-inflammatory gene signature [35]. This feature is, however, rapidly modified over time, and the pro-inflammatory gene expression is replaced by the expression of tissue-remodeling genes [35]. Additionally, very few T cells from NHP granulomas respond with cytokine production after a specific antigen restimulation [47]. Furthermore, stronger T cell responses are elicited in RMs infected with a low, rather than a high, dose of Mtb [48]. These three results are highly suggestive of immunosuppression in the TB granulomas. Following Mtb infection in NHPs, the expression of IDO was significantly induced in the myeloid layer of TB granulomas [35,45], proportional to the Mtb burden [26]. IDO appeared to be primarily expressed on IFN-responsive, interstitial macrophages in the lungs of Mtb-infected RMs [37]. While SIV-induced reactivation of Mtb infection in RMs did not correlate with the depletion of CD4^+^ T cells in the lung compartment [10], it significantly correlated with the recruitment of interstitial macrophages to the lung [24]. Thus, a direct correlation between IDO, SIV-induced reactivation, and pathology exists. IDO products were detected in TB patients in cohorts around the world [37,38,39,40], including in patients that were HIV-co-infected [28]. In fact, in South Africa, People Living with HIV (PLHIV) with active TB disease appeared to have the highest peripheral levels of IDO activity, followed by those with active TB but no HIV co-infection, and by PLHIV who had no evidence of TB disease [28]. TB treatment reduced IDO activity in individuals with TB disease in South Africa, but to an extent lower than in the country of Georgia, presumably because some individuals in the former cohort were PLHIV [28]. IDO is also expressed in the lungs of infected mice as well as in Mtb-infected macrophages ex-vivo [36,49]. The induction of IDO in these model systems correlated with the lung bacterial burden [26]. The expression of IDO, a potent immunosuppressor, is therefore induced by Mtb infection in an ordered spatial context in the immunosuppressed environment of TB granulomas.

Inhibition of IDO adjunctive to therapy: a unique opportunity to improve host responses. Host-directed therapy for TB. The interest in the concept of host-directed therapies (HDTs) or immunotherapies for TB [50,51,52,53,54,55] is driven by the desire to shorten the length of the conventional TB therapy in order to reduce the incidence of MDR-TB [56], as well as by the knowledge that effective immune responses are subverted during TB [57]. Several studies in the past decade highlighted promising candidates that either increase the effectiveness of the host in killing Mtb or reduce the destructive nature of an over-exuberant host response [49,56,58,59,60]. The IDO-mediated catabolism of the essential amino acid Trp can result in an effective innate immunity-mediated control of many intracellular pathogens [61,62,63]. Granuloma-resident Mtb can, however, synthesize Trp [44,64,65]. The host’s strategy to reduce Trp via IDO is therefore ineffective during Mtb infection. Furthermore, the Kyn metabolites of IDO impair phago/lysosome fusion and autophagy, which can kill intracellular Mtb [33,66]; inhibit the function of CD4^+^ T cells by expanding Tregs and MDSCs; deplete Trp, which is essential for the rapidly proliferating T cells during an infection; and reduce the levels of the key anti-Mtb molecule indole propionic acid (IPA) [67,68]. Together, these processes create an immunosuppressive environment conducive to Mtb persistence [26,69]. Thus, IDO blockade is an attractive HDT target for TB therapy. 

Chronic immune activation and reactivation of Mtb infection by SIV (HIV). RM are a very good model of Mtb infection, especially because of their variable response to the infection, which leads to the development of either active TB or LTBI [70]. Additionally, RM are also a very good model of Mtb/HIV co-infection, using simian immunodeficiency virus (SIV) as a surrogate for HIV [10,11,71]. Co-infection with HIV increases the risk of progressing to TB by >20 fold in people with LTBI, compared to the HIV-naïve population [72], and this can be effectively modeled in RMs [10,11,14,15,71]. In RMs co-infected with a high dose of SIV after the establishment of LTBI, most, but not all, animals reactivate the Mtb infection within a couple of months. Both reactivators and those RM which do not reactivate Mtb following SIV co-infection experience comparable CD4 T cell depletion in the periphery as well as in the lungs and are characterized by the presence of comparable viral loads [10]. These results suggest a CD4^+^ T cell-independent mechanisms of control of HIV-induced reactivation in humans. It is well known that the immune control of Mtb infection in co-infected individuals is not fully restored by ART [14,15]. HIV/SIV infections cause chronic immune activation by a combination of a persistent expression of viral proteins, aberrant inflammatory responses, as discussed earlier, including IDO [34], bystander activation of T and B cells due to proinflammatory cytokines, and microbial translocation [73]. These events result in the dysregulation of T cell function and homeostasis [74]. The infection of RMs with SIV also induces chronic immune activation in the gut [6,75]. Signatures of chronic immune activation can also be observed in the lungs of Mtb/SIV-co-infected RMs, consistent with extensive SIV [24]/HIV [76] infection being present in the lungs. IDO expression is induced in the lungs of Mtb/SIV-co-infected RMs during immune activation. Co-infected reactivators, but not non-reactivators, express lung signatures of chronic immune activation [11]. These include the upregulation of type I IFN signaling and the downstream IFN response [11]. The expression of the aforementioned IDO, which is induced in response to type I IFN, is also induced only in the reactivators, reaffirming our notion that IDO expression in the lungs during Mtb infection is directly linked to the bacillary burden [26]. The expression of IDO downstream of type I IFN [34] was localized to recently recruited, inflammatory interstitial macrophages, via a single-cell RNAseq approach [37]. The presence of a population of IDO-, IFI/IFIT-, and CXCL9-11-expressing IMs in the lung correlates strongly with TB reactivation [37]. Hence, we believe that IDO provides a link between chronic immune activation and TB reactivation.

ART alone does not completely resolve chronic immune activation during Mtb/SIV co-infection. As mentioned above, Mtb/SIV co-infection models have been developed in different NHP species [71]. Our group was the first to develop models to treat Mtb/SIV-co-infected macaques with human-equivalent ART. ART decreases the incidence of ATB in HIV-infected individuals [77] and remains the cornerstone of HIV care. However, the incidence of TB in HIV-co-infected individuals remains 4- to 7-fold higher after ART than in HIV-uninfected people in TB-endemic settings, regardless of the duration of ART or the attainment of high CD4 counts, indicating that the immune control of Mtb infection is not fully restored by ART [78,79,80]. While ART reduces inflammation and immune activation [81], the reduction is neither uniform nor complete [82], and adverse effects of ART are well documented [83], the molecular basis of which is now being investigated [84].

**Figure 1:**
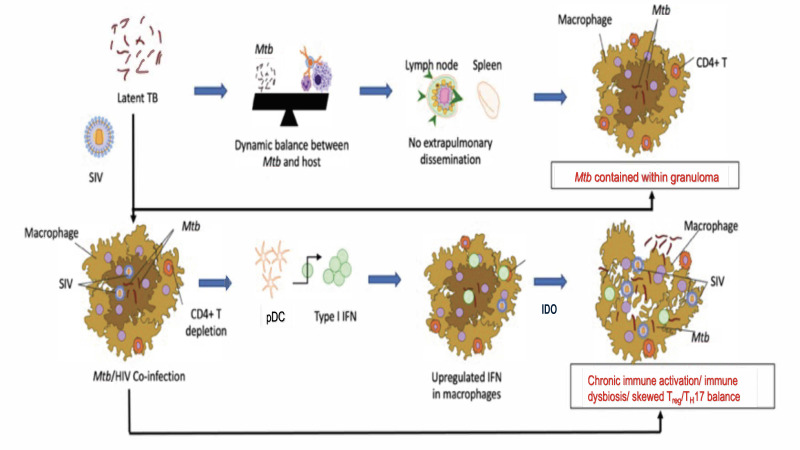
An updated working model of how type I IFN and IDO signaling could contribute to chronic immune activation and immune dysfunction during Mtb/SIV co-infections in macaque lungs.

During our NHP studies, we made interesting discoveries. Initiating ART at a time when signs of TB reactivation were already apparent did not lead to any control of TB reactivation, despite an effective control of viral replication in the periphery, alveoli and lungs and despite the reconstitution of CD4^+^ T cells [14]. The initiation of ART earlier (2 weeks post-SIV infection, as compared to 4 weeks post-SIV infection), not only resulted in the control of viral replication and CD4^+^ T cell reconstitution, but also allowed the control of the clinical reactivation of LTBI to TB [15]. Even in this group of RMs, however, the chronic immune activation was not completely controlled [15], thus mimicking the situation of Mtb/HIV-co-infected people who remain susceptible to TB reactivation despite ART. Our group earlier showed that inducible bronchus-associated lymphoid tissue (iBALT) or granuloma-associated lymphoid tissue (GrALT) are associated with protection from TB [85] and recently provided conclusive evidence for this effect [20]. Interestingly, SIV co-infection significantly depleted iBALT/GraLT, which were not reconstituted by ART. Instead, areas where iBALT/GraLT are typically located were characterized by the presence of IDO^+^ interstitial macrophages [14]. Thus, in addition to the depletion and dysfunctionality of CD4 T cells, SIV co-infection induced chronic immune activation and aberrant signaling in Mtb-infected RMs, which together drove reactivation of TB. It is important to note, however, that these studies were conducted in the absence of any anti-tubercular therapies (ATT). These results suggest that concurrent ART/ATT may be necessary to fully control chronic immune activation in co-infected RMs and, possibly, humans. Such experiments are currently ongoing in our labs. However, the mechanistic basis for how chronic immune activation contributes to TB reactivation in the context of ART and the role of IDO as a mediator of these processes needs investigation. Based on published studies, we hypothesize that SIV induces aberrant IDO signaling and chronic immune activation via induced type I IFN signaling, leading to the loss of Mtb control and the progression to TB disease. Our model (Figure 1) takes into account the fact that HIV/SIV infection induces chronic immune activation and a progressive dysfunction of anti-Mtb immunity, which is not fully restored by ART alone. This causes the recruitment of inflammatory, interstitial macrophages to the lungs [37] and macrophage turnover in Mtb/SIV co-infection [24], causing TB reactivation. We hypothesize that chronic immune activation drives immune dysfunction and the reactivation of LTBI in Mtb/SIV-co-infected RMs with dysregulated IDO signaling as a major outcome of chronic immune activation. The inhibition of IDO activity would be therefore an important tool to control immune activation and TB reactivation.

Inhibition of IDO in the NHP model of TB. The inhibition of IDO was initially attempted in SIV-infected macaques [86]. Boasso and colleagues initially determined that IDO activity was increased in SIV-mono-infected RMs [86,87], which is consistent with the data from Collins et al. in PLHIV from South Africa that we discussed in the preceding section [28]. When the IDO inhibitor D-1MT was administered, a partial reduction in IDO enzymatic activity was observed, and this coincided with a reduced viral replication synergistically with ART [86]. In vivo inhibition of IDO activity was attempted by our group successfully in Mtb-infected RMs, and more recently, as an HDT adjunctive to ATT [36]. Blockade of IDO activity with D-1 methyl-tryptophan (D1MT, Indoximod), which is currently in clinical development for cancer therapy, controlled the TB disease, significantly improved the immune responses and modified the granulomas, leading to a greater T cell influx to the lesion cores [26]. In real life, however, IDO inhibitors are unlikely to be approved for use as a TB monotherapy. Instead, if effective, they would have to be used in combination to anti-TB therapies. Therefore, we tested the effectiveness of D1MT in enhancing Mtb killing by a suboptimal treatment (Moxifloxicin + Ethambutol, ME, for 12 weeks) [36]. RMs were either not treated, so that ~50% of them developed active TB or treated with ME or with ME+D1MT (the latter for four weeks). IDO inhibition improved the immune responses and adjunctively enhanced the chemotherapeutic potential of ME. The inclusion of D1MT in the ME treatment also inhibited IDO enzymatic activity in this experiment, which was measured by immunofluorescence. Thus, the high levels of Kyn+ cells in BAL before treatment began declined after treatment, with a significantly greater reduction in the ME/D1MT group vs. the ME group [36]. Both groups of RMs, which received the ME treatment had significantly lower levels of TB disease as measured by the serum CRP levels and the change in body weight [36]. The ME+D1MT animals showed a better control of Mtb infection, with complete clearance of the bacilli relative to the ME group and harbored significantly more uninfected lungs and granulomas than the ME group [36]. The efficacy of the ME and ME/D1MT regimens were also examined using PET-CT scans as the primary correlate of progression of Mtb infection, as we previously described [43]. Using CT scans, it is possible to longitudinally count the number/size of individual granulomas; PET scans allow identifying the volume of each granuloma over time, as well as their avidity (the extent of 18-FDG incorporation), expressed either as standardized uptake values (SUV) or as total amount of absorbed radioactivity in the region of interest. All RMs had clear lungs prior to infection and focal nodular lung opacities pre-treatment. The scans performed at later stages of the experiment revealed the presence of a progressive infection in the controls, the partial effectiveness of the ME regimen and a greater effectiveness of the ME+D1MT treatment [36], suggesting that IDO inhibition is a viable adjunctive HDT strategy for TB.

IDO inhibition in Mtb/SIV co-infection and the intersection of the IDO pathway with other important immunometabolic networks. The results discussed in the preceding sections have positive implications for experiments conducted with both Mtb/SIV-co-infected RMs and Mtb/HIV-co-infected individuals. While only a handful of experiments have been performed in Mtb/SIV- (or SIV/Mtb)-co-infected NHPs, modeling the human co-infection, emerging data from these model systems suggest a critical role for type I IFN [11] and IDO, downstream of it [26], in ablating productive immunity in the lungs. These experiments suggest that IDO expression is induced in the lungs of co-infected NHPs and that in the lungs of reactivating animals, lymphocytes are replaced with IDO^+^ myeloid cells. Further, these reactivating animals are characterized by the presence of high levels of type I IFN.

Alterations in NAD^+^ metabolism are critical for host–pathogen interactions in the presence of Mtb infection [88]. Mtb infection increases the expression of CD38—a marker of inflammation—on T cells [89,90], which encodes an NAD^+^ glycohydrolase [91]. In addition to activating the host NAD^+^-degrading enzyme CD38, Mtb encodes an NAD^+^ hydrolase that is released into the cytoplasm of host macrophages, thus inducing the necrotic death of the host cells and enabling its own escape [92]. Mtb is protected from this tuberculosis necrotizing toxin (TNT)-induced cell death, since it encodes a protein inhibitor that binds and inactivates TNT in its surroundings, until TNT is released into the host cell cytoplasm [92]. While apoptotic death is considered an adaptive response to limit pathogen viability and spread, the ability of Mtb to induce necrosis reduces the presentation of Mtb antigens and likely contributes to Mtb persistence. Necrosis also avoids the consequences of apoptosis, which is more favorable to the host, leading to the destruction of the intracellular content, as well as phagocytosis and immune stimulation by antigen -resenting macrophages and dendritic cells. Thus, increasing NAD^+^ through salvage pathway precursors may prevent necrotic host cell death and potentially stimulate Mtb-specific immune responses. Low NAD^+^ levels, on the other hand, may favor the survival of Mtb. It is therefore possible that therapeutic approaches that result in an increase in intracellular NAD^+^ levels could adjunctively promote a better control of pathogens like Mtb and SIV, especially in the context of co-infection. 

The NAD^+^ and IDO–kynurenine pathways are linked, as the latter represents the de novo pathway for NAD^+^ synthesis. Earlier studies showed the potential utility of inhibiting tryptophan catabolism by IDO inhibition [26]. There is, however, a downside to IDO therapy, that is, the inhibition of the de novo NAD^+^ synthesis via Kyn as an unintended consequence. It is likely that such inhibition accounts for the compensatory, counter-regulatory, and counter intuitive results that were obtained in some studies using IDO knockout mice [93]. Considering the major intersections of the IDO and the NAD^+^ pathways, it is also possible that NAD^+^ precursors will synergize with IDO inhibitors in the control of Mtb infection. Furthermore, the IDO pathway is also connected to the signaling by Sirtuin1 (SIRT1) and AMP-activated protein kinase (AMPK) [94]. Sirtuins are a family of NAD^+^-dependent protein deacetylases involved in the control of Mtb and are activated by AMPK [95]. There is considerable evidence that diabetic persons are at high risk of being infected with Mtb and progressing to TB [96]. The mechanism of action of metformin, a drug that is used in the treatment of type-2 diabetes (T2D), involves the activation of AMPKs, which in turn, serve to activate sirtuins, which are protein deacetylases that control inflammatory cytokine activity, inflammation, and aging. In view of the increased pathogenesis of Mtb in the setting of T2D, as well of commonalties in the disease process, metformin is considered as was validated as a potential host-directed drug in the setting of Mtb infection [96]. Studies showed that many key immunometabolic circuits involved in the cross-talk with anti-Mtb immune responses are mainly controlled by the sensors mTOR (mechanistic target of rapamycin), AMPK (AMP-activated protein kinase), and SIRT1 (sirtuin 1) [97]. NAD replenishment could enhance the activity of SIRT1 in the presence of Mtb infection, where NAD levels were reduced, and further activate additional sirtuins. Mtb infection reduced AMPK and SIRT1 expression as well as their activity in macrophages and the treatment with AMPK (i.e., metformin) or SIRT1 activators (i.e., resveratrol) enhanced macrophage anti-mycobacterial function in vitro and reduced the bacterial load and TB immune pathology in a mouse model of TB [96]. Thus, the convergence of metabolic pathways and TB susceptibility in addition to the observed benefits of metformin treatment in the setting of T2DM/Mtb infection highlights exciting targets for therapeutic intervention. Thus, it is quite evident that the combination of NAD^+^ precursors with SIRT1/AMPK activators should provide for optimum NAD^+^ levels and the control of Mtb replication in vivo. Further, NAD^+^ replenishment could not only optimize the effect of IDO blockade but also synergize with SIRT1/AMPK activators to control Mtb infection.

It may be possible to inhibit IDO activity by D-1MT in Mtb/SIV-co-infected RMs on ART, with and without ATT, in order to answer the question as to whether this checkpoint inhibitor plays a key role in mediating the immunosuppressive effects arising from type I IFN induction due to chronic immune activation. It may be possible to inhibit IDO activity via multiple and diverse strategies. As discussed in the preceding sections, IDO is induced by SIV infection as well [86]. Treatment of SIV-infected RMs, without and with ART, with the active anti-inflammatory compound THC resulted in a significant inhibition of IDO via the cannabinoid receptor 2 pathway. It may therefore be possible to increase the effectiveness of IDO blockade in Mtb/SIV-co-infected RMs by concurrently treating them with D1MT/THC.

## 2. Conclusions

New approaches are urgently needed to treat the TB/HIV syndemic. Defining the molecular mechanisms that govern the reactivation of TB in the presence of Mtb/HIV co-infection is a critical research priority for developing improved therapeutic regimens in combination with ART [98]. Many critical questions, however, remain unanswered, including the fact that ART does not eliminate the risk of ATB, even when the CD4 counts are high, and the viral loads are low/undetectable. The mechanistic basis for compromised T cell functions and are the immune mechanisms beyond those dependent on CD4 T cells that mediate the breakdown of the immune control and drive TB are poorly understood. While, thus far, our work has suggested that signatures of chronic immune activation and immunosuppression (e.g., IDO) are associated with SIV/HIV progression, the underlying molecular mechanisms still remain unclear. Specifically, the role of IDO signaling/Tryptophan (Trp) catabolism in driving immune dysfunction appears to be critical and must be clarified. It remains to be seen if the blockade of IDO will improve TB disease outcomes in the absence or presence of ART in Mtb/SIV-co-infected RMs. Another important question arises from the fact that both HIV/SIV and ATT are known to induce dysbiosis in patients. Whether blocking IDO1 function reduces dysbiosis and inflammation and improves anti-Mtb responses remains to be seen. However, if these results could be obtained, as expected, then IDO blockade has the potential to improve the condition of both PLHIV and Mtb/HIV individuals through multiple effects.

The NHP model of TB, HIV, and Mtb/HIV co-infection has been instrumental in advancing our knowledge to this level, especially with respect to the immunosuppressive nature of the granuloma. Answering the critical questions highlighted in this review would allow us to gain a deep mechanistic understanding of complex immune networks and pathways in lung compartments, which is virtually impossible to do in humans but can be attempted in the NHP model. NHPs, including RMs, are particularly amenable to such studies. The ability to administer ART and block specific host pathways in vivo offers an unprecedented and unique opportunity for obtaining mechanistic insights, with translational potential for TB/HIV treatment.

## Data Availability

Not applicable
